# Epitope Peptide-Based Predication and Other Functional Regions of Antigenic F and HN Proteins of Waterfowl and Poultry *Avian Avulavirus Serotype-1* Isolates From Uganda

**DOI:** 10.3389/fvets.2021.610375

**Published:** 2021-06-15

**Authors:** John Bosco Omony, Agnes Wanyana, Kizito K. Mugimba, Halid Kirunda, Jessica L. Nakavuma, Maxwell Otim-Onapa, Denis K. Byarugaba

**Affiliations:** ^1^Department of Microbiology and Biotechnology, Uganda Industrial Research Institute, Kampala, Uganda; ^2^College of Veterinary Medicine Animal Resources and Biosecurity, Makerere University, Kampala, Uganda; ^3^Mbarara Zonal Agricultural Research and Development Institute, National Agricultural Research Organization, Mbarara, Uganda; ^4^Directorate of Science, Research and Innovation, Ministry of Science, Technology and Innovation, Kampala, Uganda

**Keywords:** *Avian Avulavirus serotype*-1, genotype-matched vaccine, B-cell epitopes, Uganda, Newcastle disease

## Abstract

Uganda is a Newcastle disease (ND) endemic country where the disease is controlled by vaccination using live LaSota (genotype II) and I_2_ (genotype I) vaccine strains. Resurgent outbreak episodes call for an urgent need to understand the antigenic diversity of circulating wild *Avian Avulavirus serotype-1* (AAvV*-*1) strains. High mutation rates and the continuous emergence of genetic and antigenic variants that evade immunity make non-segmented RNA viruses difficult to control. Antigenic and functional analysis of the key viral surface proteins is a crucial step in understanding the antigen diversity between vaccine lineages and the endemic wild ND viruses in Uganda and designing ND peptide vaccines. In this study, we used computational analysis, phylogenetic characterization, and structural modeling to detect evolutionary forces affecting the predicted immune-dominant fusion (F) and hemagglutinin-neuraminidase (HN) proteins of *AAvV-1* isolates from waterfowl and poultry in Uganda compared with that in LaSota vaccine strain. Our findings indicate that mutational amino acid variations at the F protein in LaSota strain, 25 poultry wild-type and 30 waterfowl wild-type isolates were distributed at regions including the functional domains of B-cell epitopes or *N*-glycosylation sites, cleavage site, fusion site that account for strain variations. Similarly, conserved regions of HN protein in 25 Ugandan domestic fowl isolates and the representative vaccine strain varied at the flanking regions and potential linear B-cell epitope. The fusion sites, signal peptides, cleavage sites, transmembrane domains, potential B-cell epitopes, and other specific regions of the two protein types in vaccine and wild viruses varied considerably at structure by effective online epitope prediction programs. Cleavage site of the waterfowl isolates had a typical avirulent motif of ^111^GGRQGR'L^117^ with the exception of one isolate which showed a virulent motif of ^111^GGRQKR'F^117^. All the poultry isolates showed the ^111^GRRQKR'F^117^ motif corresponding to virulent strains. Amino acid sequence variations in both HN and F proteins of *AAvV-1* isolates from poultry, waterfowl, and vaccine strain were distributed over the length of the proteins with no detectable pattern, but using the experimentally derived 3D structure data revealed key-mapped mutations on the surfaces of the predicted conformational epitopes encompassing the experimental major neutralizing epitopes. The phylogenic tree constructed using the full F gene and partial F gene sequences of the isolates from poultry and waterfowl respectively, showed that Ugandan ND aquatic bird and poultry isolates share some functional amino acids in F sequences yet do remain unique at structure and the B-cell epitopes. Recombination analyses showed that the C-terminus and the rest of the F gene in poultry isolates originated from prevalent velogenic strains. Altogether, these could provide rationale for antigenic diversity in wild ND isolates of Uganda compared with the current ND vaccine strains.

## Introduction

*Avian Avulavirus serotype-1* (*AAvV-1*) is a member of the genus *Avulavirus* in the family of *Paramyxoviridae* that causes ND in poultry. The antigenic serotypes evolving in this group under environmental or vaccination pressure, evade the immune system of birds and account for vaccine failures (1, 2). The *AAvV-1* serotype displays a great genetic and antigenic diversity in the fusion (F) and hemagglutinin neuraminidase (HN) genes that encode for the structural envelope spike proteins playing host recognition, infection, and pathogenesis roles, but importantly influencing the antigenicity and immunogenicity of the ND viruses (3–6).

Infectivity assays of ND viruses employ the use of animal models (1-day-old *Gallus gallus* chick) which requires a lot of time and monetary inputs. Low-virulent strains are defined by the monobasic amino acid sequences at positions 112–113 and 115–116 of the C-terminus of fusion protein cleavage site (FPCS) with leucine (L) at position 117 and/or intracerebral pathogenicity indices (ICPI) of ≤ 0.7 (7, 8). Such strains induce antibody titers sufficient to prevent ND with the infected birds frequently remaining asymptomatic or presenting mild respiratory form of ND. The FPCS together with the hypervariable regions of the F protein are the basis of phylogenetic analysis of pathotypes (9). Genotypes and lineages emerge among other typing methods that classify NDV strains (10, 11). To date, two classes I and II, are recognized and each further subdivided into three different subgenotypes (1.1.1, 1.1.2, and 1.2) and 21 clades or subgenotypes (I-XXI), respectively (9, 10, 12, 13). In order to avoid discrepancies of nomenclature systems and incorrect assigning of genotypes, the most recent classification emphasizes the use of full F gene to classify these viruses further (6, 12, 14). Notably, the class II genotype VII viruses are the most commonly reported in ND outbreaks in poultry, pet, and wild birds throughout the world, while class I which is commonly isolated from waterfowl, shore birds, and some poultry is less virulent and is exploited for potential vaccine candidates (10).

The potential targets for immune system response to ND virus during ND infection are the two membrane-anchored glycoproteins F and HN, also known for cell-binding and infection (6, 15). Genomic and antigenic differences between wild isolates and vaccine strains are among the reported causes of ND vaccine failure. These differences accrue from several cumulative mutations at the F and HN genes of the wild strains in response to vaccination pressure (1, 13, 16). Enzymatic restriction of the viral precursor F_0_ molecule by the host enzymes in the respiratory and intestinal tracts facilitates replication of virulent viruses in these tissues (17). The conformational change induced in the precursor F_0_ protein after proteolytic cleavage and the suspension of two disulfide-linked subunits of the hydrophobic N-terminus of F1 and the C-terminus of F_2_ fragments off the membrane initiates the cellular fusion interaction. Thus, structural protein changes produced by point mutations involving one or two amino acids or the changes resulting from recombination at the F protein antigenic site of these viruses circulating in an endemic wild birds or poultry are implicated in ND outbreaks (13, 16, 18).

In ND pathogenesis, HN initiates infection while F-glycoprotein mediates viral attachment and penetration into host cells (19). Both proteins induce host-immune response and are required for producing neutralizing antibodies induced by vaccines. Antibodies against F proteins have been demonstrated in *in vivo* study as importantly necessary in neutralizing the ND infectivity (20, 21). In fact, the passive immunization of chicks using antibodies directed to the two antigenic sites I and II of F protein, which reacted with antibodies 83(F) and 313(F), respectively, have demonstrated complete suppression of viral growth and death of chicks (22). Seven major F protein neutralizing epitopes involved in fusion inhibition and neutralization are shown at specific residues 72, 74, 75, 78, 79, 157–171, and 343 for epitopes A1, A2, A3, A4, and A5, respectively. The amino acid residues show that both F_1_ and F_2_ are involved in the formation of a single antigenic site vital in the structure and function of the active F epitopes (23, 24). In the HN protein, five conformational and linear antigenic sites are crucial in inducing antibodies at amino acid residues of 193–201, 345–353, 494, 513–521, and 569 (25, 26). Amino acid changes in the linear epitopes of wild isolates from glutamate (E) to lysine (K) at position 347 have been known to alter the character of HN protein making some strains of ND viruses escape from epitope recognition by mABs (27).

Genomic changes in the non-segmented, negative-sense RNA viruses are known to arise from the inherent error rate of polymerase, generating a large number of variants in the non-conserved proteins upon which nature selects suitable changes in the viral genome (28). Lower recombination rates are also reported in HN and F genes of non-segmented, negative-sense *AAvV-1s*, that account for strain variation (29, 30). However, strain-specific variations in *AAvV-1s*, arising from mutations are available in the public domain repositories. Nonsynonymous mutation rates have been associated with the codons at the cleavage site motif of the F protein resulting into diversity and classification of the strains. The mono- or multibasic amino acid tendencies at this site suggest that increase in virulence increases the rate of virus evolution (28, 31). Wild aquatic birds are considered to be natural reservoirs for ND viruses and constituting a threat in spreading these recombinant viruses to the domestic birds as they come into contact with them along their migration pathways across the world (32). Studies have revealed similarities between strains recovered from aquatic birds and shorebirds with those isolated from live bird markets (LBMs) in some parts of the world (10).

ND is known to be endemic in Uganda; phylogenic analysis involving F and HN sequences of wild isolates from Uganda have indicated unique ND viruses in relation to those found elsewhere in the world (33, 34). Understanding the forces of adaptive evolution or increased genetic variation in both virulent and non-virulent *AAvV-1s* can help prevent future outbreaks. Currently, two live monovalent ND vaccines (LaSota and I_2_) are available in Uganda for the control of ND infection. Due to the frequent and recent ND outbreaks, there is a renewed interest to understand the forces that drive positive or negative (purifying) pressures on specific amino acid sites within surface proteins. Importantly, the possible continued cocirculation of the virulent *AAvV-1s* in the apparently healthy-looking birds marketed at LBMs (33) needs to be studied. Recently, we isolated thermostable, low-virulent ND viruses in waterfowl and compared their immunogenicity with those of the vaccine (LaSota and I_2_) strains. We challenged the immunized birds with a circulating wild virulent strain and noted some protection failure rates (35).

Solutions to ND vaccine failure rates and the observed presence of virulent viruses harbored by healthy domestic birds can only be envisaged by establishing the role of selection pressures and virulence on evolutionary change of the NDV F protein and comparing the immune-dominant epitopes of the currently used vaccine strains with that of wild viruses using *in silico* approaches. The amino acid patterns, which are apparently hidden at sequence level become evident when mapped onto experimentally derived 3D structure. Carrying out 3D mapping of the amino acid patterns provides timely and inexpensive insights into antigen homology studies for ND control. In this study, we focused on the HN and F proteins of the Ugandan *AAvV-1s*, which are the main surface proteins important in viral entry, carrying functionally important structures like signal peptide, cleavage activation sites, and B-cell-mediated antibody response. Additionally, the sequential and conformational variations at the hydrophobic and hydrophilic sites and transmembrane sites establish the diversity of wild ND viruses and the current vaccine strains.

## Materials and Methods

### Viral Sequence Compilation

For analysis, 70 and 55 selected sequences of the F and HN protein were used, respectively. Of the 70 F protein, 40 were retrieved from the GenBank database, comprising 25 described earlier in our pathogenicity and evolution study of *Avulavirus serotype-1* isolated from poultry sampled from LBM in Uganda (33). Three ND viruses isolated from waterfowl in 2011 (36), one sequence described 10 years earlier in Uganda (34), and 11 sequences representing various class II NDV genotypes were used. The rest of the 30 sequences were partial F gene sequences of viruses isolated from freshly voided fecal matter of several species of waterfowl including the long-tailed cormorant, gray-headed gull, white-winged tern, little egret, greater cormorant, Egyptian goose, slender-billed gull, African open-billed stork, black-headed heron, gull-billed tern, African jacana, yellow-billed duck, black crake, yellow-billed stork, sacred ibis, pink-backed pelican, long-toed plover, pied kingfisher, African darter, hadada ibis, cattle egret, fish eagle, hamerkop, white-faced whistling duck, malachite, common squacco, Terek sandpiper, and wood sandpiper. Accordingly, all the isolates from LBM were highly pathogenic and of low evolution placed in genotype V in the new classification system described by Byarugaba et al. (33). Similarly, of the 55 HN protein sequences, 32 were retrieved from the GenBank database as part of our poultry LBM isolates during our pathogenicity study and evolutionary study (33) and 23 representing the 21 genotypes in class II.

The NDV isolates from poultry in Uganda were selected according to the districts, namely, Mukono (MUK), Masaka (MAS), Wakiso (WAK), Bugiri (BUG), Iganga (IGA), Kotido (KOT), Namutamba (NAM), Arua (ARU), Kiryandongo (KIR), Koboko (KOB), Nebbi (NEB), Gulu (GUL) Kasese (KAS), and Abim (ABI). The NDV isolates from waterfowl described in this study were collected from aquatic birds found at selected landing sites, namely, Musambwa Island (MUS), Makanaga Bay (MAK), Lutembe Bay (LUT), Mabamba Bay, Nakiwogo landing site (NAK), Samuka Island (SAM), and Queen Elizabeth National Park (QE) shown in Table 1.

**Table 1:** A list of Ugandan *AAvV-*1 strains and reference vaccines used in this study.

**No**	**Virus (reference)**	**Abbreviations**	**Source**	**Genotype**	**F-accession number**	**HN-accession number**
1	LaSota, vaccine strain	LaSota, vaccine strain	Vaccine^+^	II	AY845400	AY845400
2	I-2, vaccine strain	I-2, vaccine strain	Brentek^R^	II	AY935499	AY935499
3	NDV/Chicken/Pallisa/0405/01^β^	NDV/C/Pallisa/0405/01	Pallisa	II	AY367559	–
4	NDV/Waterfowl/Uganda/MU116/2011	NDV/WF/UG/MU116/2011QE	Queen Elizabeth	II	ND	ND
5	NDV/Waterfowl/Uganda/MU122/2011	NDV/WF/UG/MU122/2011QE	Queen Elizabeth	II	ND	ND
6	NDV/Waterfowl/Uganda/MU125/2011	NDV/WF/UG/MU125/2011LUT	Lutembe	II	ND	ND
7	NDV/Waterfowl/Uganda/MU126/2011	NDV/WF/UG/MU126/2011LUT	Lutembe	II	ND	ND
8	NDV/Waterfowl/Uganda/MU129/2011	NDV/WF/UG/MU129/2011LUT	Lutembe	II	ND	ND
9	NDV/Waterfowl/Uganda/MU130/2011	NDV/WF/UG/MU130/2011LUT	Lutembe	II	ND	ND
10	NDV/Waterfowl/Uganda/MU131/2011	NDV/WF/UG/MU131/2011LUT	Lutembe	II	ND	ND
11	NDV/Waterfowl/Uganda/MU132/2011	NDV/WF/UG/MU132/2011LUT	Lutembe	II	ND	ND
12	NDV/Waterfowl/Uganda/MU137/2011	NDV/WF/UG/MU137/2011LUT	Lutembe	II	ND	ND
13	NDV/Waterfowl/Uganda/MU149/2011	NDV/WF/UG/MU149/2011MAK	Makanaga	II	ND	ND
14	NDV/Waterfowl/Uganda/MU151/2011	NDV/WF/UG/MU151/2011MAK	Makanaga	II	ND	ND
15	NDV/Waterfowl/Uganda/MU152/2011	NDV/WF/UG/MU152/2011MAK	Makanaga	II	ND	ND
16	NDV/Waterfowl/Uganda/MU154/2011	NDV/WF/UG/MU154/2011MUS	Musambwa	II	ND	ND
17	NDV/Waterfowl/Uganda/MU159/2011	NDV/WF/UG/MU159/2011MUS	Musambwa	II	ND	ND
18	NDV/Waterfowl/Uganda/MU162/2011	NDV/WF/UG/MU162/2011MUS	Musambwa	II	ND	ND
19	NDV/Waterfowl/Uganda/MU165/2011	NDV/WF/UG/MU165/2011MUS	Musambwa	II	ND	ND
20	NDV/Waterfowl/Uganda/MU167/2011	NDV/WF/UG/MU167/2011MUS	Musambwa	II	ND	ND
21	NDV/Waterfowl/Uganda/MU170/2011	NDV/WF/UG/MU170/2011MUS	Musambwa	II	ND	ND
22	NDV/Waterfowl/Uganda/MU171/2011	NDV/WF/UG/MU171/2011MUS	Musambwa	II	ND	ND
23	NDV/Waterfowl/Uganda/MU172/2011	NDV/WF/UG/MU172/2011MUS	Musambwa	II	ND	ND
24	NDV/Waterfowl/Uganda/MU173/2011	NDV/WF/UG/MU173/2011MUS	Musambwa	II	ND	ND
25	NDV/Waterfowl/Uganda/MU174/2011	NDV/WF/UG/MU174/2011NAK	Nakiwogo	II	ND	ND
26	NDV/Waterfowl/Uganda/MU176/2011	NDV/WF/UG/MU176/2011NAK	Nakiwogo	II	ND	ND
27	NDV/Waterfowl/Uganda/MU178/2011	NDV/WF/UG/MU178/2011NAK	Nakiwogo	II	ND	ND
28	NDV/Waterfowl/Uganda/MU181/2011	NDV/WF/UG/MU181/2011NAK	Nakiwogo	II	ND	ND
29	NDV/Waterfowl/Uganda/MU177/2011	NDV/WF/UG/MU177/2011NAK	Nakiwogo	II	ND	ND
30	NDV/Waterfowl/Uganda/MU138/2011^e^	NDV/WF/UG/MU138/2011MAK	Makanaga	II	LT549451	ND
31	NDV/Waterfowl/Uganda/MU150/2011^e^	NDV/WF/UG/MU150/2011MAK	Makanaga	II	LT549452	ND
32	NDV/Waterfowl/Uganda/MU186/2011^e^	NDV/WF/UG/MU186/2011SAM	Samuka	II	LT549453	ND
33	NDV/Waterfowl/Uganda/MU118/2011	NDV/WF/UG/MU118/2011QE	Queen Elizabeth	II	ND	ND
34	NDV/Chicken/Uganda/MU001/2011	NDV/C/UG/MU001/2011MAS	Masaka	Vd	HG937567	HG937535
35	NDV/Chicken/Uganda/MU007/2011	NDV/C/UG/MU007/2011MAS	Masaka	Vd	HG937568	HG937536
36	NDV/Chicken/Uganda/MU009/2011	NDV/C/UG/MU009/2011MAS	Masaka	Vd	HG937569	HG937537
37	NDV/Chicken/Uganda/MU010/2011	NDV/C/UG/MU010/2011MAS	Masaka	Vd	HG937570	Hg937538
38	NDV/Chicken/Uganda/MU0138/2011	NDV/C/UG/MU013/2011MUK	Mukono	Vd	HG937571	HG937539
39	NDV/Chicken/Uganda/MU019/2011	NDV/C/UG/MU019/2011WAK	Wakiso	Vd	HG937572	HG937541
40	NDV/Chicken/Uganda/MU024/2011	NDV/C/UG/MU024/2011ABI	Abim	Vd	HG937573	–
41	NDV/Chicken/Uganda/MU026/2011	NDV/C/UG/MU026/2011BUG	Bugiri	Vd	HG937574	HG937543
42	NDV/Chicken/Uganda/MU032/2011	NDV/C/UG/MU032/2011IGA	Iganga	Vd	HG937575	–
43	NDV/Chicken/Uganda/MU033/2011	NDV/C/UG/MU033/2011IGA	Iganga	Vd	HG937576	–
44	NDV/Chicken/Uganda/MU035/2011	NDV/C/UG/MU035/2011IGA	Iganga	Vd	HG937577	HG937547
45	NDV/Chicken/Uganda/MU037/2011	NDV/C/UG/MU037/2011KOT	Kotido	Vd	HG937578	–
46	NDV/Chicken/Uganda/MU039/2011	NDV/C/UG/MU039/2011KUM	Kumi	Vd	HG937579	HG937548
47	NDV/Chicken/Uganda/MU040/2011	NDV/C/UG/MU040/2011KUM	Kumi	Vd	HG937580	–
48	NDV/Chicken/Uganda/MU044/2011	NDV/C/UG/MU044/2011NAM	Namutamba	Vd	HG937581	–
49	NDV/Chicken/Uganda/MU050/2011	NDV/C/UG/MU050/2011ARU	Arua	Vd	HG937582	–
50	NDV/Chicken/Uganda/MU056/2011	NDV/C/UG/MU056/2011ARU	Arua	Vd	HG937583	HG937552
51	NDV/Chicken/Uganda/MU062/2011	NDV/D/UG/MU062/2011ARU	Arua	Vd	HG937584	–
52	NDV/Chicken/UgandaMU069/2011	NDV/C/UGMU069/2011KIR	Kiryandongo	Vd	HG937585	HG937554
53	NDV/Chicken/Uganda/MU071/2011	NDV/C/UG/MU071/2011KIR	Kiryandongo	Vd	HG937586	HG937555
43	NDV/Chicken/Uganda/MU074/2011	NDV/C/UG/MU074/2011KOB	Koboko	Vd	HG937587	HG937557
44	NDV/Chicken/Uganda/MU084/2011	NDV/C/UG/MU084/2011NEB	Nebbi	Vd	HG937588	HG937560
45	NDV/Chicken/Uganda/MU090/2011	NDV/C/UG/MU090/2011GUL	Gulu	Vd	HG937589	HG937561
46	NDV/Chicken/Uganda/MU111/2011	NDV/C/UG/MU111/2011KAS	Kasese	Vd	HG937590	HG937566
47	NDV/Chicken/Uganda/MU113/2011	NDV/C/UG/MU113/2011KAS	Kasese	Vd	HG937591	–

*The table presents 25 poultry isolates for which full F or HN gene or both were previously sequenced and retrieved from the GenBank database using accession numbers indicated (33), 30 isolates from waterfowl for which partial F gene was sequenced targeting the F cleavage site, including*

e *Three of waterfowl F genes retrieved from the GenBank database (36)*,

β *One of the retrieved F gene of the chicken isolate was described 10 years earlier in Uganda (34). NDV, Newcastle disease virus; WF, waterfowl; C, chicken; ND, not done; M, Makerere University; UG, Uganda*.

### Sampling, Isolation, Confirmation, and Sequencing of Waterfowl *AAvV-1s* Isolates

Sampling, virus isolation, confirmation, and sequencing of the waterfowl *AAvV-1* isolates were done as described by Byarugaba et al. (33) and Wanyana et al. (36). Samples of freshly voided fecal matter were swabbed using sterile Dacron swabs from land/rock surfaces into cryovials containing viral transport medium supplemented with antibiotics (isotonic PBS, 2,000 U/ml penicillin, 2 mg/ml streptomycin, 50 μg/ml gentamycin, 50 U/ml nystatin, and 0.5% BSA). The sample vials were transported in dry shippers to the laboratory and stored at −80°C until analysis. Each sample was inoculated (in triplicate) by the allantoic route into 9–10-day embryonated chicken eggs (ECEs) for virus isolation according to the OIE Manual of Standards for Diagnostic Tests and Vaccines (37). Hemagglutinin inhibition tests, specific RT-PCR test, RNA extraction, cDNA synthesis, and sequencing were made on the harvested allantoic fluid (AF) accordingly. The AF, harvested 3 days post-inoculation was tested for hemagglutination (HA) using 1% chicken erythrocytes and ND virus confirmed by hemagglutination inhibition (HI) with an in-house rabbit-generated polyclonal anti-*AAvV-1* sera as described (37). The HI-positive samples were confirmed further by RNA extraction and cDNA synthesis by Qiagen one-step RT-PCR that extracts and amplifies the F and HN genes according to the conditions described by Byarugaba et al. (33). The amplified cDNAs of the NDV-positive extracts were separated, purified on 1% Agarose gel then visualized and documented in a Bio-Rad Gel Doc XR imager. Sequencing was done by Sanger sequencing method on a 3130XL Applied Biosystems capillary sequencer at the Plateau de Genomique GeT-Purpan, UDEAR UMR 5165 CNRS/UPS, CHU PURPAN, Toulouse France. The positive samples were also biologically characterized using standard pathogenicity tests (mean death time, intracerebral pathogenicity test, and intravenous pathogenicity test described by 33). *In vivo* evaluation of immunogenicity and protection efficacy on four of these *AAvV-1* isolates was performed previously as described in Omony et al. (35). These waterfowl *AAvV-1* isolates were sequenced for partial F gene targeting the cleavage site and the hypervariable region of the F gene. No sequenced data for the HN gene of these isolates were availed for this study.

### Sequence Data and Phylogenetic Analysis

Both nucleotide and amino acid sequences of F protein were individually retrieved from the NCBI GenBank in fasta format, matching those present in viral zone UniProtKB/Swiss-Prot entries using accession numbers. These sequences were edited to equal length and aligned using ClustalW provided in Bioedit software version 7.0.9 (38). Nucleotide similarity calculated using MegAlign Pro (DNASTAR program for life sciences, version 9, Inc., Madison, WI, USA). The waterfowl partial F sequences were aligned separately and converted into protein sequences using Emboss software (EMBL-EBI). Phylogenetic analysis was performed by Bayesian methods in MEGA version X program (39) and or Geneious Prime 2020 with the NJ Kimura 2-parameter method and 1,000 bootstrap replicates. The phylogenetic analysis was done on nucleotides based on the pilot tree proposed by Dimitrov et al. (12) to maintain the tree topology and to ascertain the genotypes of the waterfowl isolates. The same nucleotide sequences were later translated into amino acid sequences in MEGA X program v10.1.8 to compare our waterfowl, poultry isolates, and vaccine (LaSota: AY845400 and I_2_: AY935499) strains at amino acid levels.

### Generation of Structural Homology Models

To determine the conserved regions, homology models were generated for the translated F and HN proteins by aligning sequences for each gene separately using multiple sequence alignment (MSA) with the aid of ClustalW as implemented in the Bioedit program version 7.0 (38) and or Jalview. The consensus regions for each protein in each group (poultry, waterfowl, and vaccine) were used in a BLAST search against the protein Data Bank (PDB), to identify the available homologs or orthologous of known proteins. Structural homology modeling was also done using tools of SWISS-MODEL modeling server and viewed by deep viewer v4.01 (40). Overall, PDB entries 3MAW and 3T1E were used as templates for the F protein and HN protein, respectively. The experimental 3D structure (3MAW) of F protein from ND virus had identity and similarity scores of LaSota (71.1 and 74.3%), waterfowl (71.6 and 74.3%), and poultry (72.3 and 74.5%), respectively. Selected templates were subjected to chimeras to replace amino acids with *Avulavirus* F and HN protein consensus sequence. Energy minimization was carried out with YASARA force field in YASARA version 18.4.24, to obtain the most stable local minimum protein conformation. Later on, correctness of models was established using PROSTAT (module in homology) and PROCHECK (41), and the candidate epitopes were analyzed by different predication tools for structurally conserved regions (SCRs), loops, and accuracy.

### B-Cell Epitopes, MHC Class I and MHC Class II Binding Predications

A portion of the immunogenic F or HN that interacts with B-lymphocyte was assess by hydrophilic and accessible propensity scale methods and hidden Markov model programmed software from Immune Epitope Database IEDB analysis resource (http:www.iedb.org/). For linear B-cell epitopes, BepiPred was used with default threshold value of 0.4. Surface accessibility was predicted by Emini surface accessibility tool of IEDB while the antigenicity sites by the Kolaskar and Tongaonker antigenicity method with a default threshold of 1.042 (42). Peptide binding to the MHC class I and MHC class II molecules were assessed by the IEDB tools at http://tools.iedb.org/mhci/ and http://tools.iedb/mhcii/ respectively with comparison with HLA alleles by artificial neural network (ANN), stabilized matrix network (SMN), or NetMHCII pan in addition to consensus method from combination libraries. Epitope length set as 9 mers of conserved epitopes that bind to HLA at a score ≤ 1.0 percentile rank were selected.

### Mapping of Mutations, Variable Positions, and Specific Functional Regions

Following MSA, sequence variation was mapped onto the protein structures and entropy calculations with the aid of Scop3D, a tool, which visualizes variations across multiple sequences on the protein structures (43). F protein and HN protein numbering was based on LaSota using GenBank accession numbers AY845400 and P35743, respectively. The functional regions were defined based on literature. All functional regions were mapped on to the structures and Jalview or Chimera-analyzed models for diversity as visualized to the predicted structure models. Also using aligned sequences, single nucleotide polymorphisms (SNPs) were detected by trimming all gene reads and assembled to LaSota (AY845400) using Geneious assembler in Geneious Prime 2020 program (version11.0.4). During the analysis, phylogenetic tree of isolates based on selected functional regions were considered using Jalview.

### Determination of F or HN Protein Recombination Among Isolates

F and HN nucleotide sequences were separately aligned using MegaAlign software (DNAStar, 5.01) and analyzed for recombination using the split decomposition method in seven local statistical recombination tools (RDP4, GeneConv, BootScan/RecScan, MaxChi, Chimera, LARD) integrated in the RDP4 program version 4 (44). These tools were used to estimate any recombination event, recombination hotspot, recombination rate plots, etc., and to detect any putative recombination breakpoint. These methods were applied using the following parameters: window size = 20, highest acceptable *p*-value < 0.001, and Bonferroni correction. For reliable results, any four putative recombination events were detected and translated into corresponding amino acid sequences.

#### Availability of Data and Materials

F and HN sequences of the isolates analyzed in this study were all retrieved from the GenBank database with indicated accession numbers including three waterfowl isolates reported by Wanyana et al. (36) with accession numbers LT549451–53. These included NDV/WF/UG/MU138/2011 representing the 22 isolates with identical sequences, NDV/WF/UG/MU150/2011 and NDV/WF/UG/MU186/2011 provided in Table 1. Others were the 27 F gene waterfowl isolates characterized for fusion cleavage sites and were not deposited in the GenBank database.

#### Animal Ethics

The College of Veterinary Medicine Animal Resources and Biosecurity Higher Degrees Research Committee and the Uganda National Council of Science and Technology (approval #HS 776) approved this study.

## Results

### Phylogenetic Analyses Based on the Fusion Gene of *AAvV-1s*

**Figure 1:**
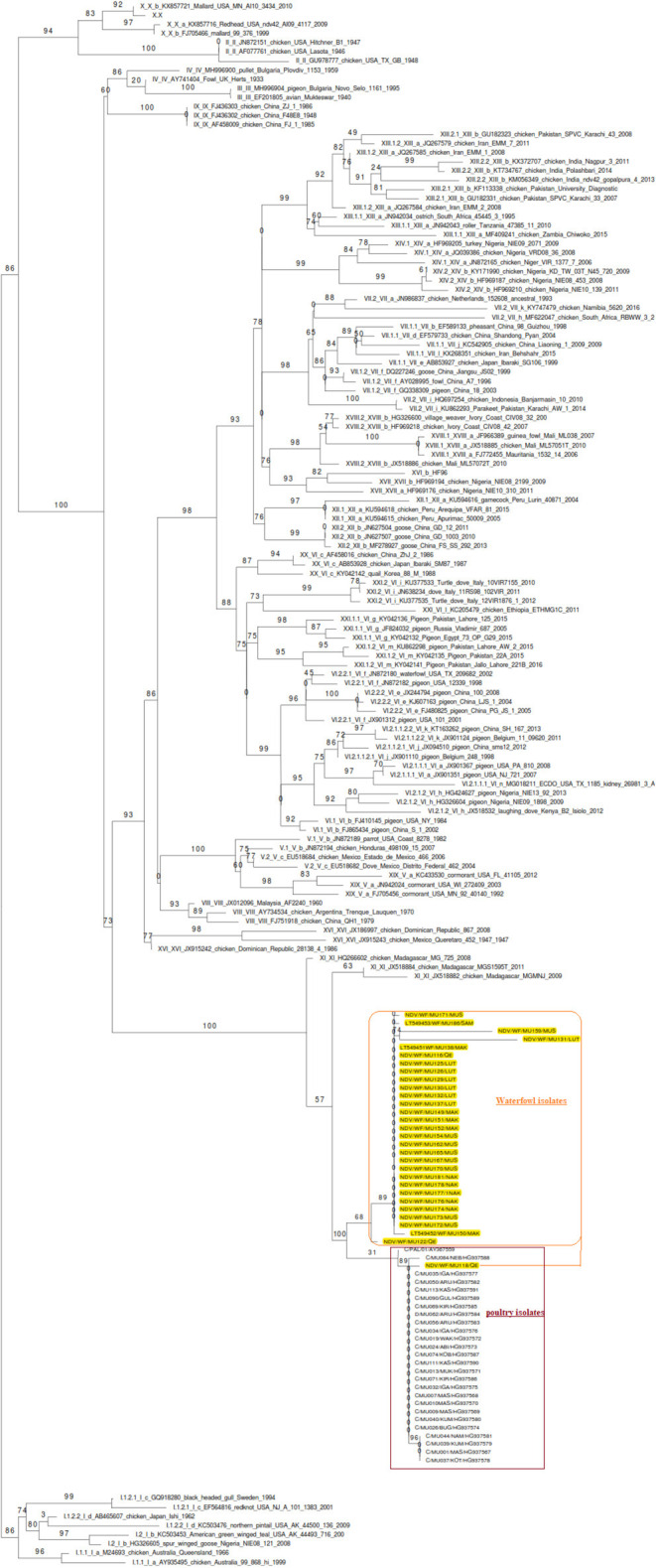
Molecular phylogenetic analysis based on the nucleotide sequences of the F gene of ND viruses of class II by neighbor-joining method. The inferred ancestral F gene sequences of the Ugandan *AAvV-1* isolates from waterfowl, poultry, and known vaccine strains based on the Tamura-Nei model using the bootstrap consensus of 1,000 replicates. *AAvV-1*isolates from waterfowl clustered differently from those of the poultry already assigned to genotype V except for one virulent waterfowl isolate (NDV/WF/MU0118/2011). The isolates from waterfowl and poultry (highlighted) did not cluster together with any of the vaccine genotypes.

The phylogenetic analyzes was performed on the full F gene (1,659 nucleotides) encoding for full F protein (553-amino acids), including the hypervariable and cleavage site regions of partial F gene (603 nucleotides) of isolates from waterfowl, encoding for a 201-amino acid protein. In order to compare the genetic relatedness of NDV isolates from waterfowl and poultry used in this study to the vaccine strains and other NDV genotypes, the phylogenetic analysis was done by comparing our isolates with the already known F sequences of class II genotype. The analyses inferred two subclusters of *AAvV-1* isolates from poultry and waterfowl. These virus isolates clustered differently from the commonly used vaccine strains and historic strains available in the GenBank database. Notably, the phylogenetic tree clustered *AAvV-1* isolates from waterfowl (*n* = 29) differently from genotype II strains, which had been historically described as genotype II. This was in exception of the virulent strain NDV/WF/MU0118/2011 isolated from Queen Elizabeth (QE) park, which clustered together with isolates from poultry (*n* = 25). Overall, the *AAvV-1* isolates from waterfowl clustered differently from the isolates derived from poultry, which were recently subtyped in genotype Vd as shown in Figure 1.

### Predicted F Protein 3D Structure, Functional and Antigenic Domains

The complete translated fusion protein amino acid sequences from poultry isolates spanning cDNA coding sequence (CDS) of 553 amino acids and the partial fusion protein from waterfowl virus isolates of 182 amino acids were used to predict 3D fusion structure and to assess the functional and antigenic domains relative to LaSota vaccine strain. Comparing these sequences at hypervariable region, proteolytic cleavage site (F_0_), N-terminus of the fusion protein (F2), cleavage motif, transmembrane regions, and amino acid involved in neutralization sites yielded differences between wild isolates and the vaccine strain. The hypervariable regions of the fusion peptide had the expected three-heptad repeat regions HRa, HRb, and HRc spanning at positions 143–185, 268–299, and 471–500, respectively. Eight variable transmembrane domains were observed between isolates from poultry and LaSota strain at amino acid positions; 14–27, 15–25, 118–131, 120–128, 266–269, 429–432, 499–525, and 501–523. Notable were substitutions in the cleavage site (*n* = 3), fusion peptide (*n* = 7), and the heptad repeat regions (*n* = 22) compared with LaSota vaccine. The change of amino acid from glycine (G) to a basic amino arginine (R) or lysine (K) at specific positions of 112 and 115, respectively, together with presence of phenylalanine (F) at position 117 affects the fusogenic activity of the F protein. This change in all the poultry isolates stabilizes the F protein allowing the host-cell protease to recognize and cleave F_0_ necessary for virulence in cells. These details are provided in Table 2. Deduced amino acid sequences of the F gene cleavage site (F_0_) in all domestic poultry isolates contained multiple basic amino acid residues. The conserved amino acid residues ^112^R-R-Q-K-R^116^, with phenylalanine at position 117 between C-terminus of F_2_ and N-terminus of F_1_ fragment of this gene confirmed the poultry isolates to belong to a virulent group of viruses. All the waterfowl *AAvV-1* isolates had the conserved amino acid residues ^112^G-R-Q-G-R'L^117^ at the cleavage site with the exception of one NDV118/WF/UG/2011QE. This was a characteristic of the avirulent strains like the vaccines strain currently in use in the country.

**Table 2:** Analysis of mutations in the F protein structure between LaSota vaccine and poultry/waterfowl isolates mapped on the 3MAW-Chain A model at hypervariable regions, fusion protein, cleavage site, neutralizing epitopes, and glycosylation domains.

**Fusion protein structure domain**	**Variable positions in fusion protein structure**																			
	**Hypervariable regions/signal peptide (1–31) and predicted antigenic region (7–30)**																	**ANT**		
AA positions	4	8	9	11	13	14	16	17	18	19	20	21	22	26	28	29	30	32	44	69
3MAW_A chain	–	–	–	–	–	–	–	–	–	–	–	–	–	–	–	–	–	–	**V**	M
Chicken structure	K	R	I	A	**L**	**T**	**I**	**T**	**Q**	**I**	**T**	**L**	**T**	**V**	**M**	**T**	S	L	**I**	M
LaSota (AY845400)	R	K	N	A	**M**	**M**	**T**	**I**	**R**	**V**	**A**	**L**	**V**	**I**	**P**	**A**	N	I	**V**	L
Waterfowl structure	K	R	I	V	**L**	**T**	**I**	**T**	**Q**	**I**	**T**	**V**	**T**	**V**	**M**	**T**	S	L	**I**	L
**Domain**	**ANT**		**Cleavage site (112–116)**		**Fusion protein (117–142)**					**HRa (143–185)**						**Antigenic region (196–241)**		**HRb (268–299)**	
AA positions	82	106	112	115	117	118	121	124	135	145	146	159	190	192	195	203	231	232	269	286
3MAW_A chain	E	–	–	–	–	–	–	–	–	N	Q	T	F	N	Q	T	T	**Q**	**I**	**Q**
Chicken structure	E	A	R	K	**F**	**V**	**V**	**S**	V	N	K	A	F	N	R	T	T	**Q**	**V**	**Q**
LaSota (AY845400)	D	V	G	G	**L**	**I**	**I**	**G**	I	K	Q	A	L	K	Q	A	N	**K**	**I**	**R**
Waterfowl structure.	E	V	G	G	**L**	**I**	**I**	**G**	I	K	Q	A	–	–	–	–	–	**–**	**–**	–
**Domain**	**HRb (268–299)**		**Antigenic region (315–332)**		**Antigenic region (380–394)**					**Antigenic region (413–437)**					**Antigenic region (447–460)**		**HRc (471–500)**	
AA positions	288	292	312	330	341	364	385	386	402	403	421	422	430	442	451	453	457	476	479	482
3MAW_A chain	**T**	V	K	**I**	T	N	**T**	L	A	D	R	H	D	A	**L**	**S**	**V**	N	D	E
Chicken structure	**N**	I	K	**L**	S	S	**A**	L	A	D	K	H	D	V	**L**	**T**	**V**	S	D	A
LaSota (AY845400)	**T**	V	R	**I**	T	S	**T**	I	V	N	K	Q	G	V	**Q**	**S**	**I**	N	N	E
Waterfowl structure	–	–	–	–	–	–	–	–	–	–	–	–	–	–	–	–	–	–	–	–
**Domains**	**HRc (471–500)**		**Major transmembrane domain (501–521)**																
AA positions	486	489	494	508	509	513	516	520	548	550	552	553								
3MAW_A chain	S	D	–	–	–	–	–	–	–	–	–	–								
Chicken structure	N	N	**N**	**A**	**V**	**L**	T	**V**	K	A	R	T								
LaSota (AY845400)	R	D	**K**	**T**	**I**	**V**	L	**I**	R	T	K	M								
Waterfowl structure	–	–	–	–	–	–	–	–	–	–	–	–								
**Domains**	**Potential B-cell epitopes^T^ (157–171)**																			
AA positions	157	158	160	161	162	164	165	167	168	196	170	171								
3MAW_A chain	S	I	A	T	N	A	V	E	V	T	D	G								
Chicken structure	S	I	A	T	N	A	V	E	V	T	D	G								
LaSota (AY845400)	S	I	A	T	N	A	V	E	V	T	D	G								
Waterfowl structure	N	M	P	P	K	P	G	K	G	I/P	N	E								

*Variable positions found in the fusion protein mutated in poultry and waterfowl compared with LaSota vaccine virus strain—AY845400 used as a reference. Bold positions are surface-exposed residues in the variable situation predicted by discrimination of protein secondary structure class. Underlined positions contribute to the hydrophobic stability of the protein. ANT, antigenic region*.

T *All these mutations were in only two waterfowl isolates but conserved in other waterfowl and in all poultry isolates*.

**Figure 2:**
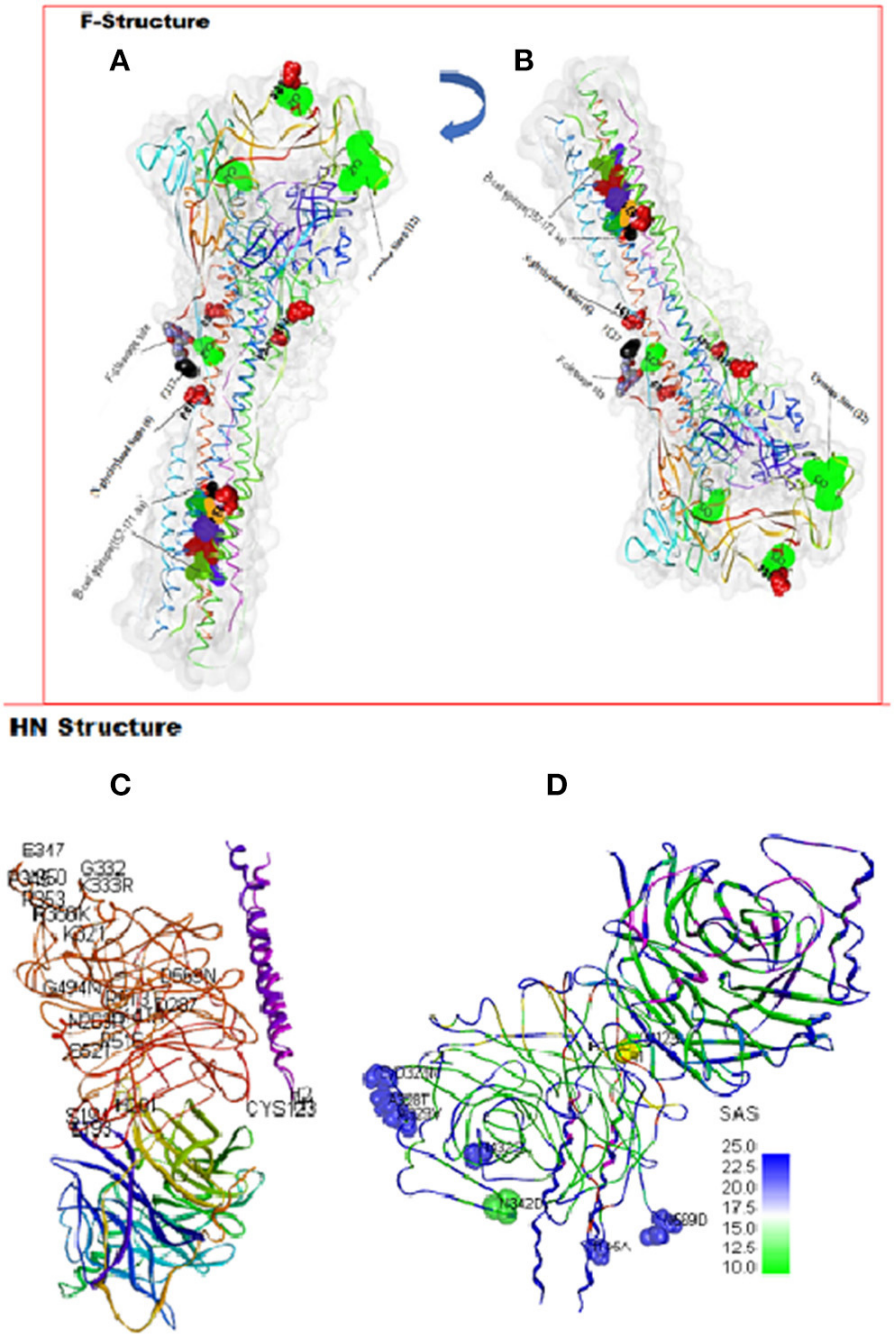
Amino acid substitutions in the predicted F and HN proteins. A PDB-3maw model for predicting variations on LaSota vaccine strain on chain A represented by a ribbon. The *N*-glycosylation, cysteine, cleavage site, and variable B-cell epitopes are labeled. The structures **(A)** and **(B)** are different orientations of the same F protein molecule rotated as shown. A PDB-3T1E chain A model HN protein ectodomain. **(C)** One dimer of the HN neuraminidase (NA) domain flanks the 2HB in the stalk side-on view packing, conserved amino acids, and substitutions positions at the antigenic sites on the A-chain are indicated. **(D)** Side-on view packing of HN dimer rotated showing the Cys-123 (yellow), Helices 1 and 2 (H_1_ and H_2_), and eight substituted amino acids (blue/green) in the antigenic sites of the A-chain displayed by solvent accessibility surface (SAS).

In both poultry and waterfowl F proteins, the six potential glycosylation sites were conserved. These sites had the motif asparagine (N)-X-serine (S)/threonine (T) where X is any amino acid except of proline (P) and aspartate (A). These six asparagine-linked glycosylation sites were at 85NRT, 191NNT, 366NTS, 447NIS, 471NNS, and 541NNT. Although the 12-cysteine residues reported at 25, 27, 76, 199, 338, 347, 362, 370, 394, 399, 401, and 424 were conserved (Figure 2A), some of them were not available for the partial F protein analyses in waterfowl virus isolates. Compared with the representative vaccines used in Uganda, the CDS of poultry and waterfowl NDV isolates, had a number of mutations at the fusion peptide (117–142), hydrophilic region A (143–185), hydrophilic site B (268–299), hydrophilic region C (471–500), and transmembrane domains of F protein (Table 3). The substitution especially at the I508A, I509V, V513L, I516V, L517T, and I520V in the transmembrane site suggests limited conservation at this site in the F gene.

**Table 3:** Comparison of amino acid substitutions within the functional domains of fusion protein sequences of Ugandan *AAvV-1* and the historical vaccine strains.

**Strains**	**Fusion peptide 117–141**						**HRa 143–185**		**HRb 268–299**		**HRc 471–500**						**Transmembrane domain (TM) 500–521**						
Amino acid positions	117	118	121	124	135	139	145	146	286	288	476	479	482	486	489	494	508	509	513	514	516	517	520
All genotypes (Cons.)	F/L	I	I	G	I	A	N	Q	Q	N	N	D	A	S	D	K	T	V	V	F	I	L	V
NDV strain LaSota	L	–	–	–	–	–	K	–	R	T	–	N	E	R	–	–	–	I	–	–	–	–	I
B1 vaccine strain	L	–	–	–	–	–	K	–	–	T	–	N	E	R	–	–	–	I	–	–	–	–	I
F-strain	L	–	–	–	–	–	K	–	–	T	–	N	E	–	–	–	–	I	–	–	–	–	–
NDV strain I2	L	–	–	–	–	S	–	–	–	T	–	–	E	–	–	–	–	–	I	C	–	–	–
V4	L	–	–	–	–	S	–	–	–	T	–	–	E	–	–	–	–	–	–	C	–	–	–
Mukteswar	L	–	–	S	–	S	–	–	–	T	–	–	E	–	–	R	–	–	–	L	M	–	–
All waterfowl isolates (Cons.)	L	–	–	–	–	–	K	–															
All chicken isolates (Cons.)	F	V/I	V	S	V	–	–	K	–	–	S	–	–	N	N	N	A	–	L	–	V	T	–

*No change in amino acid compared with the CONSENSUS (–); comparison not done ()*.

At structural level, by mapping sequence variation in poultry isolates onto the homology model PDB-entry 3MAW built on, F protein which is the F_0_ protein for *Avulaviru*s *serotype*−*1*, > 80% sequence identity was observed. The predicted 3D structure of poultry *AAvV-1* isolates was similar to that of reference LaSota (RSMD, Cα = 3.203). To map these variations onto the PDB model and perform entropy calculations, we used Scop3D and Chimeras for sequence manipulations and final visualization was done in Discovery Studio Visualize v20.1.0. We confirmed that the seven residues involved in virus neutralization from a stretch of 157–171 and individual residues D72, E74, A75, K78, A79, and L343 were conserved across all poultry virus isolates compared to LaSota. Two *AAvV-1* isolates from waterfowl; NDV/WF/MU131/LUT and NDV/WF/MU159/MUS showed 13 amino acids variability at a stretch of 157–171 site with a shift from more hydrophobic to hydrophilic amino acid residues seen in LaSota/poultry and to the two waterfowl isolates respectively (Table 3; Figures 2A,B).

We predicted linear B-cell epitopes and their surface accessibility and antigenicity scores using the BepiPred linear Epitope prediction method, Emini surface accessibility tool, and Kolaskar and Tongaonkar antigenicity, which use epitope scores and immunogenicity predictions through IEDB online (www.iedb.org) facilities. Both BepiPred-2.0 prediction tool and Vaxijen 2.0 tool gave 13 effective B-cell epitopes for LaSota vaccine and 11 for chicken isolates. Two effective epitopes at position 271–293 and 451–458 were undetected in chicken. Overall, these gave seven nonoverlapping sites above antigenicity score (0.8) and surface accessibility score (0.6); _33_DGR_35_, _164_AVHE_167_, _222_GPQITSP_228_, _223_PQITSP_228_, _407_IISQNYG_413_, _407_IISQN_411_, and _483_ESN_485_. All these were conserved in all the vaccine strain, poultry and waterfowl ND virus isolates except for the two waterfowl isolates NDV/WF/MU159/MUS and NDV/WF/MU131/LUT having substitution at amino acid 164–167. However, structure-based antibody prediction based on DiscoTope of the F protein consensus in poultry isolates showed four B-cell epitope locations (Table 4). Linear epitopes of F consensus for poultry and waterfowl isolates at (1–182 amino acid) indicated 16 substituted positions; L32I, M68L, E82D, R112G, K115G, S/D124G, V135I, N145K, K146Q, L451Q, T453S, V457I, D479N, A482E, N486R, and N489D, suggesting that these surface-exposed amino acid residues do modify the effectiveness of the predicted epitopes thus are speculated for the antigenic differences between these wild viruses and vaccine group.

**Table 4:** DiscoTope structure-based B-cell antibody prediction of chicken F protein consensus.

**Rank**	**Location**	**B-cell epitopes**	**Score^a^**	**Immunogenicity**
1	1–12	MGSKPSTRIPVP	0.700	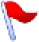
2	105–115	SVTTSGGGRQG	1.000	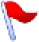
3	476–485	SALDKLAESN	1.000	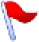
4	511–520	SLLFGVTSLV	0.808	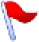

a *Score given at a default threshold (–3.7) of DiscoTope v 2.0*.

### HN Protein Structural and Functional Domains and Predicted 3D Structure

Analysis of the complete nucleotide and the corresponding protein sequences of HN from our domestic poultry *AAvV-1s* isolates, retrieved from the GenBank database was done to identify any structural differences between them to that of the available vaccine. The ClustalW MSA of the HN protein showed 93.5% identity and 97.0% similarity among the 32 domestic poultry ND viral isolate sequences. Comparing these 32 entries, all were identical except for amino acid changes at 36 positions from majority to minority shown as R3C, V9A, I27V, I34V, M35V, L37F, T48R/A, A57S, S58G, A62V, T66A, V70I, D79E, D82N, I102V, S121G, V136I, S146N, S181C, S269P, D309E, N323N, R333K, N341K, V352A, G362R, V392I, A424V, H432Y, N440S, T443I, V477I, H482R, N494D, I514T, and I548L. On sequence analysis, 77 amino acid positions showed variations between LaSota vaccine (AY845400) and the HN chicken consensus at 50% protein level. Importantly, we first focused our analysis on the structural positions of the extracellular domain consisting of surface-exposed amino acid residues since more exposed residues on the surface of the molecule influences the overall charge and antigenicity than the ambivalent or internal amino acid. Twenty-six amino acid positions were varying with LaSota vaccine observed in this category, namely, Q7R, D13E, R62A, N63I, R65K, E68D, K69R, D79E, K98S, N120T, Y203H, N263R, R269S, G293K, S310D, D342N, R356K, V387E, S432H, S440N, H474Y, Y479H, G494D, V495E, D569N, and G570K. Importantly, five of these E68D, K69R, K356R, D569N, and G570K were noted to lie within antipeptides previously used to produce monoclonal antibodies for differentiating birds infected by vaccine isolates and virulent (Exotic Newcastle disease) strains.

Expectedly, the HN protein for the domestic poultry ND virus isolates had the predicted stalk and globular head regions of the HN protein, comprising 1–143 and 125–571 amino acids, respectively. Three transmembrane domains were obtained at positions 24–47, 25–45, and 557–563. Predicted amino acid residues had the 12 cysteine (C) positions conserved at 172, 186, 196, 238, 247, 251, 455, 461, 465, 531, and 542. In addition, the reported cysteine at position 123 in certain known isolates was present and not replaced by tryptophan (W) like in some vaccine strains. The key biologically active residues for receptor (sialic acid) binding site were conserved at positions R174, E401, R416, and Y526; the hydrophobic core of the stalk 4HB was conserved at positions Y85, V88, S92, L96, T99, I103, I107, L110, and I114 together with the stalk residues at R83, A89, L90, L94, and L97 involved in direct interaction with F protein.

Organizing these variations and others into functional domains like cytosolic, transmembrane, and flanking regions are shown in Table 5. However, assembling the HN nucleotide sequences on the CDS of LaSota vaccine (AY845400), all the 32 reads predicted only four polymorphic amino acid changes that had effect on the HN protein shown as V35M (75.8%), M48V (36.4%), A66T (81.8%), and S440N (48.5%) with codon changes G103A, A142G, G196A, and G1319A, respectively. Importantly, 28 charge position variations observed were due to substitutions between the poultry isolates and LaSota strain sequences from the model (PDB-3T1E). Positions 42A → S and 43S → I are located in the transmembrane domain affecting the charge and physiochemical properties of HN protein in poultry isolates. 84I → V and 98K → S lie proximately at the hydrophobic and hydrophilic sites predicted at MHC I alleles and linked to known T-cell epitope affecting the charge of the peptide. Comparison of the HN antigen-exposed surfaces and the predicted potential B-cell epitopes of consensus sequence of isolates from poultry and LaSota strain using the BepiPred-2.0 prediction tool on IEDB server, 25 epitopes were discovered. Analysis of their antigenicity with Vaxijen 2.0 tool and accessibility with Emini Surface Accessibility Prediction tool showed 15 out of 25 effective epitopes. Six (positions: 555–566, 108–115, 526–537, 236–254, 370–381, and 147–166) out of 15 effective epitopes were similar to those in LaSota vaccine strain. The other nine epitopes (positions: 539–547, 452–481, 283–291, 493–508, 263–269, 183–213, 404–430, 437–445, and one transmembrane motif 23–48) were dissimilar in structure and epitope threshold effectiveness due to mutations at positions 203Y → H, 263N → R, 265A → V, 266V → A, 269R → S, 290T → V, 356R → K, 352I → V/A, 404I → V, 440S → N, 443T → I, 453I → V, 464P → S, 466V → I, 476L → I, 477I → V, 494G → N/D, 495V → E, 502A → V, 508S → Y, and 569D → N which all lie at the predicted conformational B-cell epitope in the 3D structure (Figures 2C,D). In addition, the linear ^343^TCPDEQDYQIRMA^355^ and ^493^DGV^495^ epitopes determining the monoclonal antibody reactivity ability of HN protein of NDVs showed mutations. Majorly the above substitutions altering the nine epitopes could be implicated in the vaccination failures while using LaSota vaccine.

**Table 5:** Variation of amino acid in the HN antigenic, transmembrane, and its flanking regions.

**Site**	**Cytoplasmic region AA (1-20)**				**Transmembrane domain/signal cleavage region (28-40) AA (21-48)**											**Antigenic site**			**Flanking region**		
Residues	4	7	9	13	29	31	33	34	35	36	38	42	43	45	48	57	58	60	62	63	65
LaSota strain	A	Q	A	D	I	F	T	V	V	T	A	A	S	L	M	V	G	P	R	N	R
B1 vaccine strain	–	–	–	–	–	–	–	–	–	–	–	–	–	–	–	–	–	–	–	I	–
F-strain	–	–	–	–	–	L	–	I	–	–	–	I	–	V	–	–	–	–	–	I	K
I2 strain	–	–	–	–	–	–	–	–	–	–	–	–	A	A	–	–	–	–	A	I	–
V4 strain	–	–	–	–	–	L	–	–	–	–	–	–	A	A	–	–	–	–	A	I	–
Komarov strain	–	–	–	–	–	L	–	–	–	–	–	–	–	V	–	–	–	–	R	I	–
Mukteswar strain	–	–	–	–	A	L	M	–	I	–	–	V	A	A	–	–	S	–	A	I	–
Chicken (50% Con.)	V	R	V	E	V	S	I	I	M	I	V	S	I	V	T	A	S	S	A	I	K
Site								Antigenic					Antigenic							Antigenic	
Residues	66	68	69	70	73	120	123	126	127	136	145	156	182	265	266	269	290	293	307	310	315
LaSota strain	A	E	K	I	T	N	W	L	I	I	A	F	A	A	I	R	T	G	F	S	S
B1 vaccine strain	–	–	–	–	–	–	–	P	–	–	–	–	–	–	–	–	–	G	–	–	–
F-strain	–	–	–	–	V	–	–	P	–	–	–	–	–	–	–	P	–	E	–	–	–
I2 strain	–	–	–	–	A	S	C	P	–	–	D	–	–	–	A	S	–	E	–	N	P
V4 strain	–	–	–	–	A	S	C	P	–	–	–	–	–	–	I	S	–	E	–	N	P
Komarov strain	–	–	–	–	A	–	–	P	–	–	–	–	–	–	–	L	–	E	–	–	S
Mukteswar strain	–	–	R	–	A	–	C	P	V	–	T	–	–	V	I	S	–	R	–	N	P
Chicken (50% Con.)	T	D	R	V	L	T	C	P	V	V	I	Y	T	V	A	S	V	K	L	D	P
Site						Ant		Ant	Antigenic site							Antigenic					
	328	329	342	369	387	404	432	440	453	464	466	474	476	477	479	484	495	502	508	540	570
LaSota strain	T	V	D	I	V	I	S	S	I	P	V	H	L	I	Y	L	V	A	S	T	G
B1 vaccine strain	–	–	–	–	–	–	–	–	–	S	–	Y	–	–	–	–	E	A	–	–	–
F-strain	–	L	–	–	–	–	–	–	–	S	–	Y	–	–	–	–	–	A	–	–	–
I2 strain	–	A	–	V	–	V	–	–	–	S	–	Y	–	V	–	–	–	V	G	–	–
V4 strain	–	A	–	V	–	V	–	–	V	S	–	Y	–	V	–	–	K	V	–	–	–
Komarov strain	–	V	–	I	–	I	–	–	I	S	–	Y	–	–	–	–	E	A	–	–	–
Mukteswar strain	–	A	–	V	–	V	N	N	V	S	–	Y	–	V	H	–	K	V	N	–	–
Chicken (50% Con.)	A	A	N	V	E	V	H	N/S	V	S	I	Y	I	V	H	V	E	V	Y	V	K

*The underline amino acid positions alter markedly the charge of the HN protein of the 32 poultry isolates analyzed. Variations at Cysteine-123 and N-glycosylation-508 positions are high-lightened*.

At structural level, the homology model of the *AAvV-1* HN protein was based on the Australia-Victoria/32 strain (PDB-3T1E chain A). Our homology model using chicken isolates consensus HN protein was similar to 3D experimental PDB model (root mean square deviations (RMSD) of Cα = 1.2A). Several positions of amino acids in the HN protein were variable between the mapped 3D LaSota and the consensus chicken isolate HN protein structures (Figures 2C,D). Structurally, NDV HN protein is a multifunctional glycoprotein playing a key role in viral infectivity. The amino acid residues R174, I175, D198, K236, E258, Y299, Y317, E401, R416, R498, R516, Y526, and E547 essential for receptor recognition, were conserved across all the 32 ND virus isolates from poultry in this study, suggesting they infect the similar hosts (ducks and chicken) from where they were isolated.

The original five *N*-glycosylation sites in the HN protein of the isolates from poultry were all conserved between themselves at positions 119NTS, 341NNT, 433NKT, 481NHT, and 538NKV but with differences in the patterns of amino acids at three positions 119NSS, 341NDT, and 538NKT when compared with LaSota and I_2_ vaccine strains commonly used in Uganda. *N*-Glycosylation reported at amino acid position 508 was absent in all our isolates from poultry but replaced by 508N → Y. Comparisons of the HN protein total length of vaccine strain with wild isolates revealed several point mutations at transmembrane domains including the hydrophobic (HRa) and hydrophilic (HRb) sites and in the six continuum of antigenic sites 2, 3, 4, 12, 14, and 23 that form 3D conformation of the HN molecule (Table 6).

**Table 6:** Variations in structurally and functionally important residues of the HN protein in poultry and vaccine strains mapped on the 3T1E-chain A model structure.

**Virus**	**Hydrophobic/hydrophilic sites**						**Antigenic neutralizing epitopes residues**																				
	**HRa**			**HRb**			**1**	**2**				**3**			**4**			**12**		**14**				**23**			
	**(74–88)**			**(96–110)**			**345**	**(513–569)**				**(363–321)**			**(332–356)**			**(494–516)**		**(347–353)**				**(193–302)**			
	**75**	**81**	**84**	**98**	**101**	**102**	**345**	**513**	**514**	**521**	**569**	**263**	**287**	**321**	**332**	**333**	**356**	**494**	**516**	**347**	**350**	**352**	**353**	**193**	**194**	**201**	**203**
Lasota	G	V	I	K	T	T	P	R	I	S	D	N	D	K	G	K	R	G	R	E	Y	I	R	L	S	H	Y
B1	–	–	–	N	–	–	–	–	–	–	–	–	–	–	–	–	–	–	–	–	–		–	–	–	–	–
F-strain	–	–	–	S	–	–	–	–	–	–	–	–	–	–	–	–	–	–	–	–	–	–	–	–	–	–	H
I2	–	–	–	N	S	–	–	–	–	–	–	–	–	R	–	–	–	D	–	–	–	–	–	–	–	–	H
V4	–	–	–	N	S	–	–	–	–	–	–	–	–	–	–	–	–	D	–	–	–	–	Q	–	–	–	H
Komarov	–	–	–	N	–	–	–	–	–	–	–	–	–	–	E	–	–	G	–	–	–	–	–	S	–	–	
Mukteswar	–	–	–	N	S	I	–	–	–	–	G	K	–	–	–	R	–	D	–	–	–	–	–	–	–	–	–
Chicken	S	I	V	S	S	I	–	–	T/I	–	N	R	–	–	–	R	K	N/D	–	–	–	V	–	–	–	–	H

### The F and HN Protein Recombination Among Isolates

Using all recombination detection methods in RPD4, there was no recombination detected while analyzing the HN gene of both isolates from waterfowl and poultry compared within, among themselves, or with vaccine strain used in Uganda. However, using F gene of individual groups or combined in the presence of LaSota, I_2_ and I_2_ progenitor as reference vaccines, there were two breakpoints that signaled the recombination events which were attributable to an evolutionary process in isolates of waterfowl using the stringent criteria of any four recombination detection methods. The seven statistical methods used in the RPD4 were automated RPD(R), GeneConv (G), Bootscan (B), MaxChi(M), Chimaera (M), SiScan (S), and 3SEQ (T). Several potential recombination points were detected; two of these in the isolates from waterfowl and one isolate from poultry are shown in Table 7.

**Table 7:** Fusion gene recombination confirmation by split decomposition method using local statistical methods.

**Detection method**	**Poultry viral isolate pairs**		
	**Recombinant NDV/C/UG/MU074/2011KOB**	**Major parent LaSota**	**Minor parent NDV/WF/UG/MU032/2011IGA**
	**Sequence detected in**		**Av**. ***p*****-value**
RDP	1		3.537 × 10^−39^
GENECONV	25		9.107 × 10^−1^
BOOTSCAN	1		0.100
MAXCHI	5		6.309 × 10^−1^
CHIMAERA	5		–
SISCAN	–		–
3SCAQ	1		7.771 × 10^−15^
**Detection method**	**Waterfowl viral isolate pairs**		
	**Recombinant NDV/WF/UG/MU131/2011LUT**	**Major parent LaSota**	**Minor parent NDV/WF/UG/MU159/2011MUS**
	**Sequences detected in**		**Av**. ***p*****-values**
RDP	1		3.282 × 10^−2^
GENECONV	4		1.850 × 10^−6^
BOOTSCAN	1		6.872 × 10^−7^
MAXCHI	3		3.588 × 10^−10^
CHIMAERA	2		2.387 × 10^−5^
SISCAN	–		–
3SCAQ	28		3.54 × 10^−15^
**Detection method**	**Recombinant NDV/WF/UG/MU118/2011MUS**	**Minor parent LaSota**	**Minor parent NDV/WF/UG/MU159/2011MUS**
RDP	1		5.367 × 10^−6^
GENECONV	1		5.978 × 10^−5^
BOOTSCAN	2		1.856 × 10^−3^
MAXCHI	2		3.72 × 10^−3^
CHIMAERA	2		2.868 × 10^−3^
SISCAN	–		–
3SCAQ	–		–

**Figure 3:**
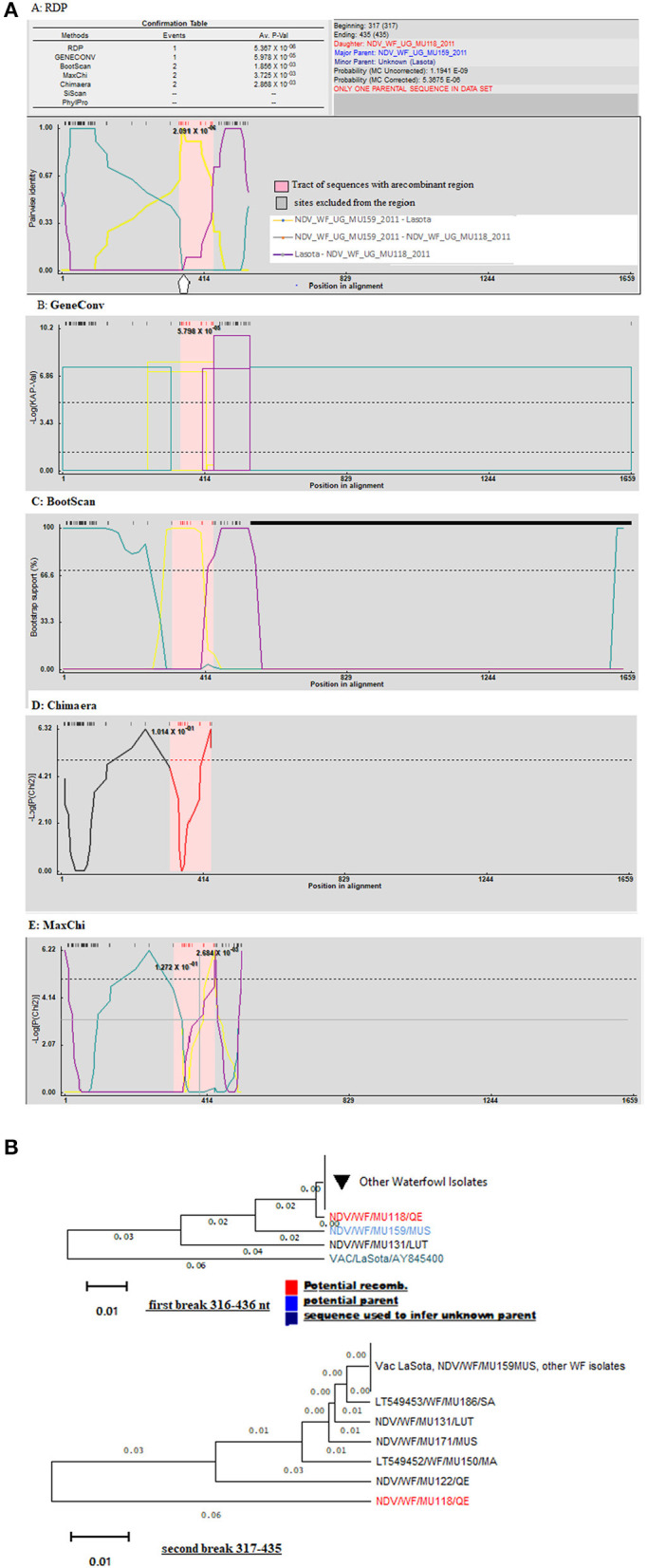
Graphic representation of recombination in fusion gene of *Avian Avulavirus*es. **(A)** Lines forming an open rectangle connects the beginning and the end of a fragment that underwent recombination. The height of the open rectangle is proportional to –log10 of the probability. **(B)** Phylogenetic trees for different regions of F gene showing the movement of recombinant fragments (red colored) in the NDV isolates from waterfowl. Trees were generated with the RDP program (version 4) by using UPGMA analysis of region derived from both major and minor parents. Bootstrap values (percentage from 1,000 replicates) were shown for the relevant nodes.

Using only GeneConv to search for the possibility of significant recombination, which uses the homologous segments, we detected few recombination breakpoints in the isolates of poultry. By using less-stringent criteria of two recombination detection methods, we found 25 statistically significant recombinant fragments in isolates from poultry, observed at lengths 11–437 nt and 439–1,659 derived from major parent (LaSota: 93.9% similarity) and 12–438 nt regions derived from minor parent (NDV/C/UG/MU032/2011IGA: 100% similarity) in the fusion gene producing a recombinant strain NDV/C/UG/MU074/2011KOB. At the same time, 30 recombinant fragments were observed in isolates from waterfowl at lengths 10–438 to 438–546 nt and 439–1,654 nt derived from minor parent (LaSota: 99.2% similarity) and major parent (NDV/WF/MU159/MUS: 100% similarity) respectively producing a recombinant strain NDV/WF/MU131/LUT. However, only two most important breakpoint events compatible with recombination with a beginning breakpoint at nucleotide positions 316 to 436 nt and 317 to 435 nt using the five recombination detection methods (Figure 3A: A–E) were identified in the F gene of waterfowl NDV isolate NDV/WF/MU118/QE isolated from a conservation area (Queen Elizabeth National park). Both FPCS and intracerebral pathogenicity indices showed the daughter virus to be virulent. They also indicated that it appeared to have arisen from a recombination between a non-virulent NDV/WF/MU159/MUS and LaSota vaccine with statistically significant probabilities (*p* < 0.01). The lines in open rectangles seen in the GeneConv plots (Figure 3A: B) connect the probable beginnings and ends of the fragments that underwent recombination.

Recombination signals were also detected between I_2_ and I_2_ progenitor vaccines and the NDV isolates from waterfowl at varying lengths along the fusion gene. GeneConv in the RDP sorted and scored all the significant fragments in the alignment and listed them in decreasing Bonferroni-corrected *p*-values. Testing this assortment of sequences provided a maximum parsimony pedigree suggestive of recombination. However, the exact point of fragmentation together with the relationships of the ND viruses waterfowl isolates involved can be identified by the tree-like network. The *p*-values produced guided in the identification of the fragments and locations of recombination as shown in Figure 3B.

## Discussion

The economic impact of ND on both the backyard and commercial poultry industry in Uganda and elsewhere is significant. The annual disease resurgence, vaccine failures, and potentially virulent strains circulating in apparently healthy birds become an issue. The concerns of viral evolution, type of vaccine used to protect flocks, and post-vaccine evaluation are always raised. This study evaluated the predicted B-cell epitopes and functional domains of F and HN proteins in *AAvV-1* isolated from Ugandan aquatic birds and poultry and compared them with those of the vaccines to determine variations between the two groups that might explain ND vaccine failures. To date, *AAvV-1* vaccine evaluation is based on empirical crossprotection of birds. Many of such studies indicate an increasing evidence that antigenically matched ND vaccines provide better immunity (45, 46). However, such studies are costly and laborious and require extended field studies. Notwithstanding other factors that might lead to vaccine failures, insufficient cold-chain maintenance, insufficient immunity titer, hygiene status, etc. have been of great concern. Here, we focused on *in silico* study of the neutralizing epitopes of HN and F-glycoproteins of endemic wild ND viruses and vaccine (genotype II) to ascertain the presence of virus variants and any recombinants at these two antigenic and surface glycoproteins in our *AAvV-1* isolates that could result into inefficient vaccination.

In Uganda, vaccination using live avirulent LaSota and recently introduced I_2_ strain, belonging to genotypes II and I, respectively, of class II offers the best ND control. Phylogenetic analysis of *AAvV-1s* reported in the two previous independent studies showed that the partial and full F gene sequences of *AAvV-*1 isolates from waterfowl (36) and poultry (33) clustered Ugandan viruses with those of genotypes II and Vd viruses, respectively. This is consistent with previous reports of a predominance of genotypes II and V viruses in wild and domestic birds. However, based on the current unified system of classification described by Dimitrov et al. (12), the subgenotype Vd becomes V due to lack of the branch support. However, phylogenetic analysis of the same viruses combined in the current study showed two subclusters; one cluster of genotype V where poultry belonged and other (unassigned) where waterfowl isolates belonged. No virus isolates from waterfowl clustered with genotype II as previously reported except that one virulent (NDV/WF/MU118/2011QE) strain clustered with poultry isolates (genotype V). Although knowledge of circulating genotypes established through phylogenetic analyses is important in epidemiology or evolutionary changes of NDV, little about the antigenic diversity at the B-cell epitopes is gained from these tree analyses, suggesting that selection of neutralizing variants arising from mutations or fragments of gene recombination implicated in vaccine failures have to be identified. Besides, the proper genotype to where the *AAvV-1* isolates from waterfowl are placed in class II viruses warrants investigation.

The importance of antibodies directed toward both F and HN epitopes that strongly neutralizes ND infectivity is important for understanding the diversity of these genes in wild virus isolates and vaccine strains. In this study, we demonstrated amino acid substitutions in the entire length of F and HN proteins in *AAvV-1* isolates from waterfowl and poultry as compared with vaccine strains used in Uganda. Analysis of the hypervariable site of F protein revealed two groups, one with multiple basic acid residues at the proteolytic cleavage site _112_RRQKR_116_ F_117_ at the N-terminus of F2 and F1, respectively, where viruses from poultry belonged. The other group had _112_GRQGR_116_L_117_ where those from waterfowl and vaccine strains belonged. This confirms the incidences of *AAvV-1s* in susceptible domestic and feral bird species and suggestive of the potential role of feral birds in transmitting these viruses to poultry. The reported (RK/RQRR↓F) motif cleavage site among *AAvV-1* isolates within the same genotype from domestic poultry and other wild hosts have been attributed to native selection, host pressure selection, viral evolution, or wide vaccination (14). There was no evidence of variation of pathogenicity among strains in paired samples isolated from cloaca and oropharynx of a single bird (33), and the presence of *AAvV-1s* with different pathogenicity based on the cleavage site motif within one geographical area suggests a long time geographical isolation of these different pathotypes. Waterfowl harboring viruses with lentogenic F-cleavage motif site generally, except for one isolate, and healthy-looking poultry harboring virulent *AAvV-1* strains confirmed by the RRQKR↓F F-cleavage motif site, suggests that the two groups differ in their fusion activity and virulence in their hosts (31). This is of great importance and should be noted by both epidemiologists and vaccinators since variability in amino acid residues at the FPCS of viruses existing in the same geographical location suggests presence of different viral genotypes and pathotypes and potential for vaccine failure (47). The presence of avirulent ND viruses in waterfowl indicates that wild birds may be a potential source of virulent *AAvV-1* for poultry due to possible point mutations at FPCS (7, 48). At the same time, these wild birds might therefore serve as a source of potential vaccine isolates (35).

Conservation of F and HN proteins at *N*-glycosylation, cysteine sites, and other specific structural and functional sites like receptor (sialic acid) binding sites between *AAvV-1* isolates from waterfowl and poultry together with vaccine strains indicate these are required for biological active state especially among amino acids residues involved at the direct interactions that form a high-level structural integrity (49, 50). The substitutions particularly in the surface-exposed amino acid residues in signal peptide (position: 13–29 aa) is not surprising considering that the amino acids in this region undergo constant positive selection making them highly variable. However, the mutations in the fusion peptide (position: 117–142 aa) is least expected except for the two nucleotides necessary for a change from a trypsin-like to a furin-like site. The exceptional changes at the nearby position of this site especially at V118I, V121I, S124G, V134I, and N145K in isolates from poultry and D124G and A129V in *AAvV-*1 isolates from waterfowl warrant further investigation in view of recombination detected in this region. The hypervariable hydrophobic regions (HRa, HRb, and HRc) especially I269V, T288N, I330L, T385A, Q451L, S453T, I457V, and K494N whose side chains are surface-exposed (51), together with T508A, I509V, V513L, and I520V of the transmembrane region of F protein are implicated in structural variations between the wild and vaccine strains. The 13 observed mutations in the F protein of the two *AAvV-1* isolates from waterfowl at the known antigenic aa position 157–171 account for variation. These favored more of hydrophilic aa residues (*n* = 5) at positions N157S, K162N, K167E, N170D, and E171G than hydrophobic aa residues (*n* = 2) at positions M158I and I169T while other aa were neutral in nature (*n* = 6). Only K167E and E171G of the hydrophilic aa decreased the protein stability by the predictor of effects of single-point protein mutation in I-Mutant Suite. Taken together, increased antigenicity and the neutralizing epitopes of F protein of these two viruses could result into altered fusion activity and neutralizing escape variants (52, 53). The implications of these changes in the critical regions of F and HN genes (neutralizing epitopes) could be serious as they may mean escape of the virus from immunity.

Structurally, the seven and five major epitopes on the F protein and HN proteins, respectively, have been mapped on to experimental 3D structure in the PDB. Our findings located these functional residues and found those of F protein to be more conserved at positions 72, 78, 79, 343, and 157–171 in all virus isolates from poultry and vaccine strains emphasizing the role of mABs elicited by this glycoprotein in the neutralization of NDV (54). However, the 13 substitutions occurring in two virus isolates from waterfowl at a stretch of 157–171 aa between LaSota strain, displaying mutations N157S, K162N, K167E, N170D, and E171G, which are hydrophilic and exposed on the outer periphery of the protein surface had improved antigenicity of these epitope which are necessary for antibody binding and are crucial for the *AAvV-1* isolate antigen diversity. This suggests cocirculation of these novel viruses similar to those identified earlier as vaccine candidates (35). On the other hand, the eight substitutions in the HN protein-neutralizing epitopes together with other functional sites between LaSota strain and *AAvV-1* isolates in this study could explain why these are antigenic variants which fail to be neutralized by antibodies produced in healthy poultry birds vaccinated with LaSota vaccine and sold in the market.

However, our previously reported vaccination results in Omony et al. (35) did not fully prevent ND infection under our experimental conditions where we used four avirulent NDV isolates from waterfowl as vaccine strains and challenged the immunized birds with a virulent NDV isolates from poultry. In any case, excretion of the challenge virus might have occurred. Not ignoring other factors of immune status of the vaccinated flocks, level of hygiene, and cold-chain maintenance to safeguard vaccine doses, protection against ND is dependent on the genotype and immunogenicity of the vaccine virus (35). Therefore, it would be important to develop an effective vaccine that is able to produce and maintain a high antibody level in vaccinated flocks. In our case, the variations in amino acid between the wild isolates and vaccine strains could be implicated in antigenic variants and vaccine failures leading to annual ND incidence.

Evolution of *AAvV-1* isolates has continuously posed threats of emergence of new virulent strains and challenges of diagnosis of ND (55). Many studies have suggested that different strains of *AAvV-1* evolve through various degrees of accumulation of point mutations and gene exchange by recombination (23). However, our analysis did not detect any recombination in the HN gene of wild *AAvV-1* isolates and from the LaSota vaccine strain, but we obtained signals of recombination in the F gene confirming previous findings in the genotype V viruses (29, 30). The virulent *AAvV-1* isolates from poultry possessing the virulent motif ^112^RRQKRF^117^ which is different from that of LaSota strain ^112^GRQGRL^117^ can be explained by the acquisition of the F2-terminus of F gene from virulent viruses retaining their avirulent F1-terminus giving a novel clade of genotype (V) of Ugandan ND-viruses. On the other hand, *AAvV-1* isolates from waterfowl maintain in nature their avirulent consensus of F1-terminus. This is again corroborated by studies that have showed that *AAvV-1* isolates from poultry highly select pathogenic derivatives of the virus from nonpathogenic precursors (56). The N-terminal of F gene and the rest of the genes in the *AAvV-1* isolated from waterfowl had high degree of similarity with those of the genotype II (LaSota strain). Therefore, it was more likely that the F2 terminal of F gene in *AAvV-1* isolates from poultry originated from one of the prevalent LaSota (genotype II) strain or isolates from waterfowl. Although it has been noted that the F cleavage site of ancestor viruses, such as the long used live LaSota vaccine strain tends to be conserved, the potential of these viruses mutating to highly virulent strains with passage in chicken is a notable concern. Our current study noted recombination at 316–436 nt region that codes for cleavage site and its surrounding F1-terminus region (106–145 aa) which produced the only virulent daughter strain among the low virulent strains. It is not surprising considering that this region was predicted as a potential B-cell epitope by structural-based DiscoTope tool in *AAvV-1* isolates from waterfowl. However, such event suggests that further work is warranted to investigate this role in the waterfowl NDV virus isolates. Recombination in F gene *AAvV-1* viruses is suggested here as an important parameter to be considered in vaccination with live, attenuated viruses. The F gene recombination in NDV might be a potential reason for immunization failure.

## Conclusion

We have evaluated *AAvV-1s* isolates originally assigned to genotypes V and II isolated from chicken and waterfowl, respectively, in Uganda. Based on the deduced amino acid analysis, structures mapping various mutations were found throughout F and HN genes of the isolates that affect the predicted B-cell epitopes; some were exclusive to either *AAvV-1* isolates from waterfowl or poultry. Recombination was also confirmed in the F gene of this non-segmented negative-strand RNA virus. These changes could have real impact on the immunity and disease dynamics of the virus in vaccinated as well as immunologically naïve flock. Mixed or closed feeding of poultry with waterfowl in NDV-prevalent areas and frequent and uncontrolled use of vaccines could have serious implications in the control of ND where vaccination was done. Whether all these changes individually or combined influence the antigenicity of the virus leading to serious consequences of vaccine efficacy need to be established.

## Data Availability Statement

The datasets presented in this study can be found in online repositories. The names of the repository/repositories and accession number(s) can be found below: https://www.ncbi.nlm.nih.gov/genbank/, LT549451-53, HG937535-91.

## Ethics Statement

The animal study was reviewed and approved by College of Veterinary Medicine Animal Resources and Biosecurity Higher Degrees Research Committee and Uganda National Council of Science and Technology (Approval #HS 776) approved this study. Written informed consent was obtained from the owners for the participation of their animals in this study.

## Author Contributions

JBO, DKB, HK, JLN, and MO-O designed the study. JBO and DKB analyzed the sequenced data. AW, JBO, and KKM collected the samples, isolated *AAvV-1* strains, and identified the viruses. All authors read and approved the final manuscript.

## Conflict of Interest

The authors declare that the research was conducted in the absence of any commercial or financial relationships that could be construed as a potential conflict of interest.
